# Quality analysis and function prediction of soil microbial communities of *Polygonatum cyrtonema* in two indigenous-origins

**DOI:** 10.3389/fmicb.2024.1410501

**Published:** 2024-05-31

**Authors:** Li Yang, Qing Yang, Jiansang Wulu, Yue Wang, Wenfang Jin, Zhigang Yan, Zhifeng Zhang

**Affiliations:** ^1^School of Pharmacy, Southwest Minzu University, Chengdu, China; ^2^Tibetan Plateau Ethnic Medicinal Resources Protection and Utilization Key Laboratory of National Ethnic Affairs Commission of the People’s Republic of China, Southwest Minzu University, Chengdu, China; ^3^National Engineering Institute for the Research and Development of Endangered Medicinal Resources in Southwest China, Guangxi Botanical Garden of Medicinal Plants, Nanning, China

**Keywords:** *Polygonatum cyrtonema*, fungi, bacteria, 16S rDNA, polysaccharides and saponins

## Abstract

*Polygonatum cyrtonema* Hua (PCH), as an important economic crop, is used as raw industrial materials and traditional Chinese medicine. There are significant variations in the quality of PCH from different geographical origins. It can be due to the change of the endophytic fungi and soil microbial communities of PCH. Therefore, the aim of this study is to investigate the composition and functional prediction of the main microbial communities in the rhizomes and soil of PCH and explore their impact on medicinal quality. High-throughput sequencing techniques targeting ITS and 16S rDNA were employed to compare the structure and biodiversity differences of endophytic fungi in the rhizomes and soil microbial communities of PCH from 12 different locations in Sichuan and Guangxi province. Heatmap analysis was used for comprehensive statistics and visualization of the richness of rhizome and soil microbial communities from all locations. Venn analysis was conducted to determine the total number of shared fungi between rhizomes and soil, and GraphPad Prism analysis was employed to predict and compare the microbial communities related to phenotypes at the genus level in Sichuan and Guangxi. Tax4Fun and Fungild were used for metabolic function prediction of microbial communities in the rhizomes and soil of PCH. The results revealed the identification of 19,387 bacterial amplicon sequence variants (ASVs) in the rhizomes and 37,990 bacterial ASVs in the soil, with 6,889 shared bacterial ASVs. In addition, 2,948 fungal ASVs were identified in the rhizomes and 8,868 in the soil, with 1,893 shared fungal ASVs. Microbial sequencing results indicated that the fungal communities between soil and rhizomes were mainly composed of *Ascomycota* and *Basidiomycota*, while bacterial communities included *Proteobacteria*, *Acidobacteria*, *Bacteroidota*, *Gammatimonadota*, and *Firmicutes*. Dominant bacterial groups such as *Nitrospira*, *Acidibacter*, and fungal groups including *Mortierella*, *Ceratobasidium*, and *Fusarium* were identified as potential contributors to the observed traits. In the top 15 microbial genera, both Sichuan and Guangxi contain 15 bacterial genera, but there are differences in their abundance. Guangxi has three unique fungal genera, including the genera *Scleroderma*, *Russula*, and *Gliocladiopsis*. On the other hand, Sichuan has the unique fungal genus *Chamaeota*. The correlation analysis between the microbiota and the chemical content from 12 different collecting spots was performed by GraphPad Prism. *Burkholderia-Caballeronia-Paraburkholderia*, *Acidibacter*, and *Amycolatopsis* show an inverse proportionality to total polysaccharides and saponins, while *Enterobacter* shows a direct proportionality to total polysaccharides and inverse proportionality to saponins. The metabolism pathways show a significant positive correlation with PCH polysaccharides and saponins. This study provide new insights into the mechanisms underlying the quality differences between the two major indigenous areas.

## 1 Introduction

*Polygonatum cyrtonema* Hua (PCH) is a perennial herbaceous plant belonging to the Liliaceae family (Gong et al., 2024). It is a traditional Chinese medicine with a history of over 2000 years, valued for its medicinal properties (Xu et al., 2023). The rhizomes of PCH are known for their effects in supplementing vital energy, strengthening the spleen, and benefiting the kidneys. Referred to as “Immortal’s Residual Food,” it is often considered a longevity-promoting health food (Liu and Si, 2018; Hu et al., 2022). The plant primarily grows in the southwestern and southern regions of China, below an altitude of 3,000 meters, in thickets, understory forests, or shaded mountain slopes (Xu et al., 2023). As wild resources decline, the PCH industry has rapidly developed, leading to a supply-demand gap. Currently, artificial cultivation is the primary source of PCH medicinal materials (Boroduske et al., 2021).

Research indicates that endophytic fungi play a crucial role in plant growth and yield. These fungi contribute to regulating plant resistance systems, especially in combating diseases and pests (He et al., 2018). Plants harbor diverse endophytic fungal communities that assist host plants in stimulating growth, restoration, and responding to various stresses (Deng and Cao, 2017). Cai analyzed the diversity of endophytic fungal communities in rhizome samples from five different locations of PCH (Lü et al., 2023b). Zhu investigated the composition and diversity of soil fungal communities in the rhizosphere of rhizome systems under four different integrated management modes (Zhou et al., 2020). While previous studies have demonstrated the association between microorganisms and the quality of PCH, there is a lack of in-depth exploration of the diversity between endophytic fungal communities and soil microbiota. The study aims to address this gap by investigating the microbial ecology of both the rhizomes and surrounding soil and to declare the relationship between the indigenous quality and microbial ecology. Our research group conducted a comprehensive evaluation of the quality of PCH medicinal materials in the early studies. The results indicate significant differences in the active ingredients and pharmacological effects of PCH medicinal materials from different growing areas, especially in the Sichuan and Guangxi provinces (Zhang et al., 2023).

Plants can alter the chemical cycling of the soil, transforming inherent and unchanging components into usable nutrients and modifying the structure and function of soil microbial communities, thereby significantly impacting subterranean biological communities (Bay et al., 2021). Simultaneously, the soil microbial community can decompose organic matter, facilitate the release of soil nutrients, propel energy transfer, and control biogeochemical cycles of the Earth, encompassing macro (micro) elements and nutritional elements (Jin and Lu, 2023). Therefore, understanding the compositional characteristics of soil microbial communities is crucial for studying the quality of plant growth, contributing to a deeper exploration of the microbial ecological mechanisms underlying variations in PCH quality.

Despite some researches have been performed in previous research on bacterial and fungal interactions (Deveau et al., 2018), data on the microbial ecology of PCH from different cultivated spot are still limited. Few researches simultaneously focused on the ecological aspects of microbial communities in both the rhizosphere of PCH and its surrounding soil (Yuan et al., 2021). We hypothesize that PCH, regardless of its geographical origin, represents a distinct habitat for specific microbial communities. The composition of microbial communities of the soil closely associated with the rhizome of PCH, may be intricately linked with the biological characters and quality of PCH (Wang et al., 2021).

The study employed 16S rDNA and ITS sequencing technologies to explore the diversity and composition of bacterial and fungal communities in the rhizomes and soil of PCH. Comparative analyses will be conducted between microbial communities from two different production areas. Functional predictions of bacteriamicroorganisms will be performed to identify pathways related to the biological characters of PCH. The study aims to provide insights into the ecological mechanisms of PCH quality variation, offering scientific data for future cultivation and quality improvement and contributing to the ecological theory and practical considerations in large-scale cultivation and selection of production areas and environmental conditions for PCH.

## 2 Materials and methods

### 2.1 Experimental design and sampling

The rhizomes of PCH and the rhizosphere soil were collected from Sichuan and Guangxi provinces, respectively (Supplementary Figure S1). The geographical locations of each sample were over 100 kilometers apart. Detailed collection information is provided in Table 1. The identification of all medicinal materials was conducted by Professor Hao Zhang from the West China School of Pharmacy, Sichuan University.

**Table 1:** Collection information for PCH sample.

Sample ID	Origin	Longitude	Latitude	Altitude/m
S1	Xuecheng Town, Li County, Sichuan Province	103°16′11.23″	31°38′41.34″	2569.36
S2	Muding Town, Yingshan County, Sichuan Province	106°48′59.41″	31°6′40.05″	405.24
S3	Huangdu Town, Yingshan County, Sichuan Province	106°45′48.13″	31°6′16.42″	237.41
S4	Wangjia Town, Nanbu County, Sichuan Province	106°9′50.40″	31°9′277.13″	356.26
S5	Gongqiao Town, Anyue County, Sichuan Province	105°5′14.83″	29°57′39.45″	367.28
S6	Zhonglong Town, Zizhong County, Sichuan Province	104°56′11.67″	29°45′15.19″	275.56
G1	Jiaojiang Yao Ethnic Township, Quanzhou County, Guangxi Province	110°51′6.04″	25°37′0.01″	289.90
G2	Rongjiang Town, Xing’an County, Guangxi Province	110°28′55.05″	25°30′35.21″	283.03
G3	Jinpen Village, Lingchuan County, Guangxi Province	110°29′24.03″	25°26′14.21″	711.08
G4	Pingdeng Town, Longsheng County, Guangxi Province	109°50′37.73″	26°12′14.04″	419.89
G5	Zhaoping County, Hezhou City, Guangxi Province	111°9′2.13″	24°18′37.04″	306.97
G6	Jinxiu Town, Jinxiu Yao Autonomous County, Guangxi Province	110°12′7.12″	24°8′54.04″	1204.44

Plants collected were 3–4 years old, with 30 randomly selected PCH plants harvested from each production area. Samples were prepared by combining specimens from six plants, and various phenotypic traits of PCH were measured, including plant height, stem diameter, leaf length, leaf width, leaf number, rhizome length, and rhizome diameter. Measurement methods included using a measuring tape for plant height, a caliper for stem diameter, and measurements of well-developed leaves at the upper, middle, and lower parts for leaf width and length (Supplementary Figure S2). The collected data were recorded in the PCH phenotype record sheet, with results recorded precisely to 0.01 cm (Table 2). Soil samples were collected around the rhsizomes of PCH within approximately 2–4 mm. Each sample was a composite of several adjacent sampling points, and soil samples were sieved through a 2 mm mesh to remove debris. The soil collection and processing followed the guidelines outlined in the《Analysis Method of Agricultural Chemistry in Soil》. For each production area, six sets of rhizomes had soil carefully removed from their surfaces, washed with sterile water, and then placed in liquid nitrogen or dry ice. Samples were transported to the laboratory on dry ice and stored at −80°C for subsequent experiments.

**Table 2:** Morphological characteristics of PCH from different regions.

Sample ID	Plant height/(cm)	Leaf length/(cm)	Leaf width/(cm)	Stem thickness/(cm)	Leaf blade (leaves)	Stem length/(cm)	Stem diameter/(cm)
S1	157 ± 6.50	18 ± 1.50	7.5 ± 1.50	0.6 ± 0.45	18 ± 4	15 ± 4.00	6 ± 3.00
S2	104 ± 7.00	15 ± 3.00	2.3 ± 1.10	0.5 ± 0.20	33 ± 9	13 ± 3.00	5 ± 2.50
S3	94 ± 8.00	14 ± 3.00	2.1 ± 0.85	0.5 ± 0.25	26 ± 9	12 ± 4.50	3 ± 2.00
S4	70 ± 7.50	4 ± 1.05	4 ± 1.25	0.5 ± 0.15	14 ± 5	10 ± 3.50	6 ± 4.50
S5	72 ± 8.00	15 ± 1.50	2.4 ± 1.00	0.5 ± 0.20	34 ± 6	12 ± 3.00	6.5 ± 3.75
S6	65 ± 6.50	12 ± 2.0	2 ± 0.50	0.4 ± 0.15	20 ± 5	10 ± 3.50	4.5 ± 1.75
G1	80 ± 60	12 ± 3.80	3.5 ± 1.45	0.6 ± 0.10	15 ± 7	8.7 ± 3.00	2.8 ± 0.60
G2	70 ± 6.50	17 ± 2.00	2.6 ± 0.75	0.3 ± 0.10	14 ± 9	8.5 ± 3.75	2.3 ± 0.85
G3	50 ± 8.50	10 ± 1.30	2.5 ± 0.55	0.3 ± 0.10	12 ± 2	7.0 ± 3.50	2 ± 1.25
G4	122 ± 6.00	14 ± 4.00	3.2 ± 1.00	0.6 ± 0.10	16 ± 5	12 ± 4.25	3.5 ± 1.0
G5	86 ± 7.50	15 ± 3.50	4.8 ± 0.60	0.5 ± 0.25	13 ± 3	9.7 ± 3.35	2.3 ± 0.60
G6	78 ± 8.50	15 ± 3.30	4.8 ± 1.80	0.4 ± 0.10	13 ± 3	10 ± 2.35	2.2 ± 0.80

### 2.2 High-throughput marker gene library preparation and sequencing

All collected samples (72 soil and 72 PCH samples) were handled under sterile conditions. Soil samples were extracted from the tubes using a steel spatula and freeze-dried before DNA extraction. Similarly, approximately 0.5 g of fungal tissue from the interior of each fresh PCH plant was transferred to a 1.5 mL centrifuge tube with a flame-sterilized scalpel and used for DNA extraction. DNA from soil and rhizomes was extracted using the dnasy Power Soil DNA extraction kit (Life Technologies, Magen) and the MagPure Soil DNA KF Kit (Cat. No. D6356-02, Magan, Thermo Fisher Scientific Inc., United States) machine, respectively. The concentration and purity of DNA were measured using a NanoDrop 2000 spectrophotometer (Thermo Fisher Scientific, Waltham, MA, United States) and agarose gel electrophoresis, and the extracted DNA was stored at −20°C. PCR amplification of the bacterial 16S gene and fungal ITS gene was performed using extracted genomic DNA as a template, specific primers with barcodes, and Takara Ex Taq high-fidelity enzyme. The primers used for 16S rDNA amplification were 343F (5’-TACGGRAGCAGCAG-3′) and 789R (5’-AAGGTATCTAATCCT-3′), and for ITS amplification were ITS1 (5’-TCCGTAGGTGAA CCTGCG G-3′) and ITS2 (5’-TCCTCCGCTTATTGATATGC-3′) (Mukherjee et al., 2014).

PCR products were assessed by gel electrophoresis, purified using AMPure XP beads, used as templates for the second round of PCR, and purified again with magnetic beads. The purified second-round products were quantified using Qubit, and concentrations were adjusted for sequencing. Sequencing was performed on the Illumina NovaSeq 6000 platform, generating 250 bp paired-end reads. Library construction and sequencing were conducted by Shanghai OE Biotech Company (Shanghai, China).

### 2.3 Bioinformatics analysis and statistical methods

The raw data were in FASTQ format. After data acquisition, cutadapt software was used to trim off primer sequences from raw data sequences. Subsequently, DADA2 (Callahan et al., 2016) was employed to perform quality filtering, denoising, merging, and chimera removal on the qualified paired-end raw data using default parameters in QIIME 2 (2020.11), resulting in representative sequences and ASV abundance tables. Representative sequences for each ASV were selected using the QIIME 2 package, and all representative sequences were annotated by alignment with databases. The Silva database (version 138) was used for 16S alignment, and the Unite database was used for ITS alignment. Species alignment annotations were analyzed using the q2-feature-classifier software with default parameters (Callahan et al., 2016; Zheng et al., 2021).

QIIME 2 software was employed for α and β diversity analyses. Alpha diversity, assessing the α diversity of samples, was evaluated using indices including the Chao1 (Chao and Bunge, 2002) and Shannon (Hill et al., 2003) indices. Before analyzing β diversity, data were normalized using cumulative sum scaling (CSS) in the metagenome Seq package. Principal coordinate analysis (PCoA) was performed using the “ordinate” function, and multivariate analysis of variance (PERMANOVA) using the “adonis” function, assessing differences between sample groups. The “betadisper”function was used to evaluate group variances. *Post hoc* pairwise comparisons were conducted for the same group of samples using the “adonis” function in the pairwise Adonis package. The “multipatt” function in the indicspecies package was used to select ASVs with the strongest associations and to obtain indicative species. The “plot_heat” function in the metacoder R package was used to generate heatmaps of the abundance of different taxa in different habitats. A balloon plot was created to illustrate the relative abundance differences of ASVs between rhizomes and soil for different fungal species in PCH. Venn diagrams were also generated, and correlations between the phenotypic traits of PCH, such as plant height and rhizome diameter, and microbial communities were analyzed using GraphPad Prism.

### 2.4 Determination of polysaccharides and saponins contents from PCH

#### 2.4.1 Instruments and chemicals

Varioskan LUX2 fully automated microplate reader (Thermo Fisher Scientific Inc., United States); HWS-26 electric constant temperature water bath (Shanghai Yiheng Scientific Instrument Co., Ltd., China); 96-well plates (Wuxi Naisi Life Science Co., Ltd., China); Eppendorf pipettes (Eppendorf AG, Germany).

Glucose and diosgenin were obtained from the National Institute for the Control of Pharmaceutical and Biological Products (Beijing, China), anthraquinone, sulfuric acid, vanillin, acetic acid, and perchloric acid are all analytical grade and were purchased from Tianjin Zhiyuan Chemical Reagent Co., Ltd. (Tianjin, China).

#### 2.4.2 PCH polysaccharides and saponins extraction and content determination

Weigh out 1.0 g of PCH separately, add 10 mL of distilled water, reflux at 95°C for 1 h, filter while hot, make up for the weight loss, and obtain the polysaccharide extract, add 10 mL of 80% ethanol to the residue, perform ultrasonic extraction at 30°C for 40 min with a power of 60 W, filter, make up for the weight loss, and collect the filtrate to obtain the saponin extract.

#### 2.4.3 Determination of polysaccharides and saponins content

Polysaccharides and saponins were detected based on previous method with some modification (Zhang et al., 2024). The anthrone-sulfuric acid colorimetric method was performed to determine the polysaccharides, and the absorbance is set at 582 nm. Saponin is determined using the 5% vanillin-acetic acid method, and the absorbance is set at 452 nm.

#### 2.4.4 Construction of glucose and diosgenin standard curve

Accurately pipette 0.2 mL, 0.4 mL, 0.6 mL, 0.8 mL, 1.0 mL, 1.2 mL, 1.4 mL, and 1.8 mL of 0.2 mg/mL diosgenin reference solution into stoppered test tubes, evaporate the solvent in a constant temperature water bath at 80°C, add 0.2 mL of freshly prepared 5% vanillin-acetic acid solution to each tube, and then add 0.8 mL of perchloric acid at 60°C with shaking, heat the mixture in the water bath at 60°C for 15 min, remove and cool in an ice bath for 2 min, add acetic acid to a final volume of 5 mL, mix well and let stand for 5 min, 1.4 Transfer 200 μL of the solution to a 96-well plate, with triplicate wells for each sample, measure the absorbance at 452 nm and plot the standard curve with absorbance on the *y*-axis and concentration on the *x*-axis.

Accurately pipette 0.1 mL, 0.2 mL, 0.4 mL, 0.6 mL, and 0.8 mL of 0.335 mg/mL glucose reference solution into 10 mL graduated test tubes, add distilled water to each tube to a final volume of 2.0 mL, mix well, and slowly add 0.2% anthrone-sulfuric acid solution in an ice bath until reaching the graduation mark, mix well and keep warm in a boiling water bath for 10 min, remove from heat and immediately cool in an ice bath for 10 min, pipette 200 μL of each sample onto a 96-well plate, with triplicate wells for each sample, measure the absorbance at 582 nm wavelength and plot the standard curve with absorbance on the *y*-axis and concentration on the *x*-axis.

## 3 Results

### 3.1 High-throughput sequencing results

After filtering, a total of 5,510,237 (average of 79,363 ± 72,855 per sample) 16S sequences and 5,381,577 (average of 78,021 ± 77,565 per sample) ITS sequences were obtained from soil samples. From rhizome samples, 5,541,286 (average of 79,363 ± 72,855 per sample) 16S sequences and 5,410,947 (average of 79,617 ± 28,690 per sample) ITS sequences were obtained. After further removal of non-target sequences and chimera reads, the total number of bacterial communities in rhizomes and soil was 4,465,710 and 3,982,608 (22,090 and 43,508 bacterial ASVs), respectively. For fungal communities, the total number was 5,119,081 and 4,887,376 (4,846 and 10,775 fungal ASVs) in rhizomes and soil, respectively.

## 4 α-diversity

**Figure 1:**
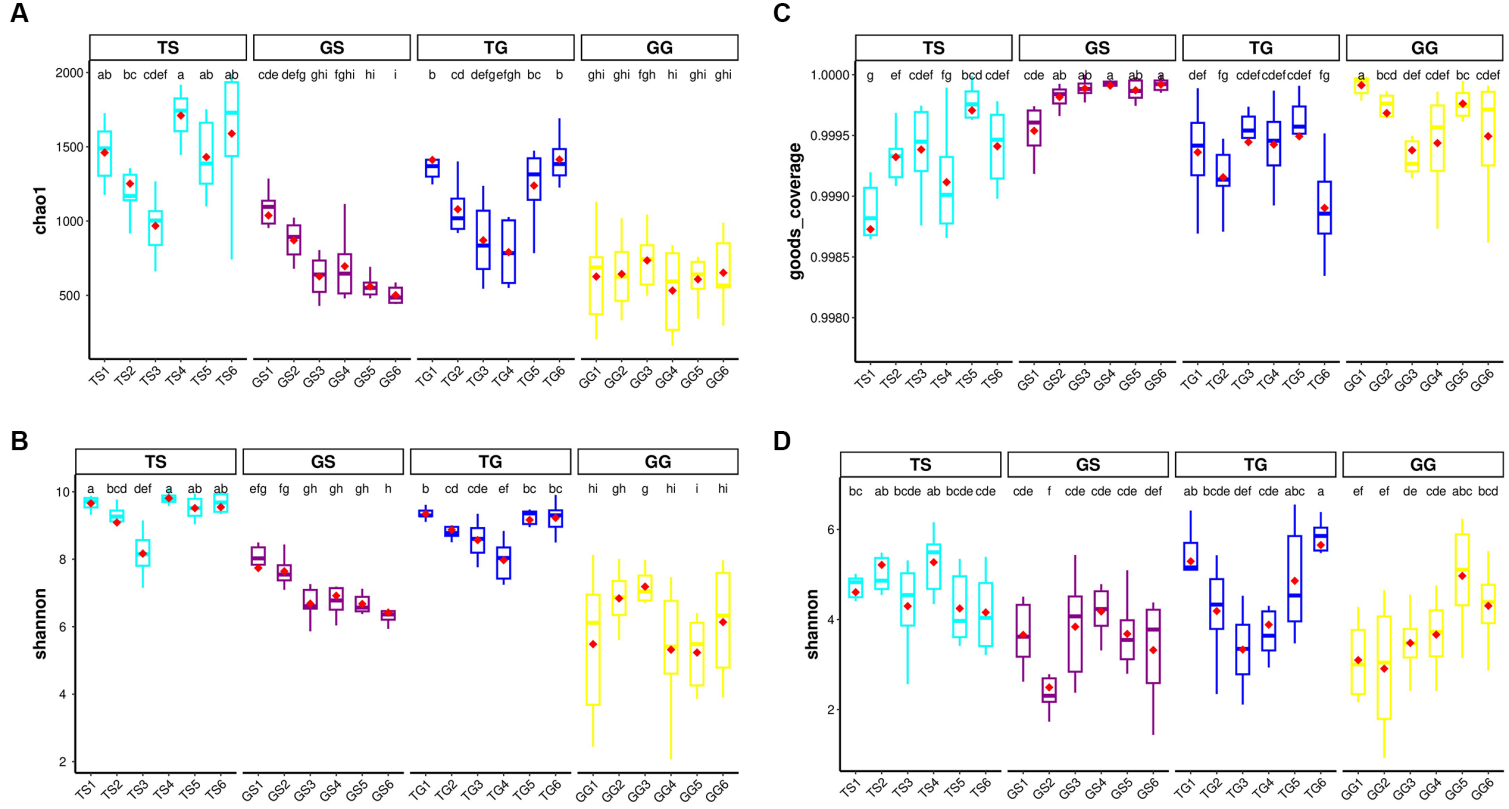
Illustrates the α-diversity boxplots of Chao1 richness and Shannon diversity indices for bacteria communities in the soil and Rhizome **(A-B)** dataset, displays the α-diversity boxplots of good-coverage richness and Shannon diversity indices for fungal communities in the soil and Rhizome **(C-D)** dataset. Subplots are grouped by location. The red diamonds represent the mean distribution, and significant groups are indicated by letters placed above the boxes.

In the dataset, the richness and Shannon index of fungi and prokaryotes within PCH were significantly lower compared to soil (*p* ≤ 0.05). However, there were significant differences between PCH samples collected from 12 different locations and soil samples. Boxplots illustrate the values of observed richness and Shannon diversity index of fungi (Figures 1C,D) and bacteria (Figures 1A,B). It is noteworthy that bacterial richness in PCH did not show a clear difference (Figures 1A,B). Supplementary Figure S3 provides dilution curves for both curves, indicating that the sequencing depth is sufficient to recover all diversity within the samples for both fungi and prokaryotes. These results suggest that the sequencing data volume is reasonable and can reflect the majority of microbial information in the samples. There were significant differences in Shannon richness in both fungi and prokaryotes in soil (Figures 1C,D). Boxplots display the values of observed Shannon and Chao1 diversity indices for fungi and bacteria in both Figure 1.

## 5 β-diversity

**Figure 2:**
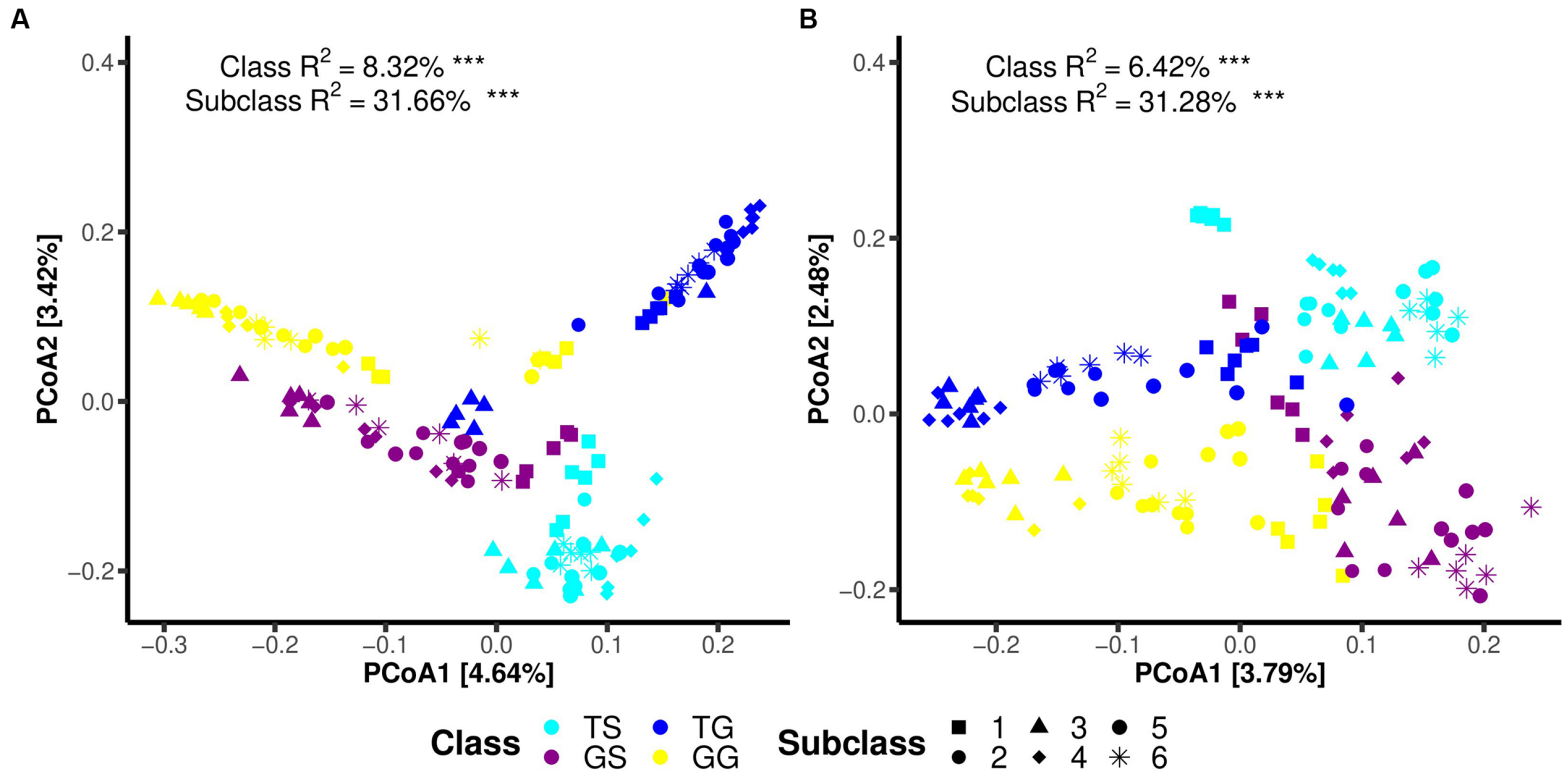
Illustrates Principal Coordinate Analysis (PCoA) plots for bacteria **(A)** and fungal **(B)** communities using the Bray-Curtis dissimilarity matrix. Each plot reports the PC values obtained from PERMANOVA analysis, with *p*-values less than 0.05, along with the R2 values obtained from adonis analysis. Significance codes for asterisks: *** indicate a significance level of 0.001, * indicate a significance level of 0.05.

Principal Coordinate Analysis (PCoA) on the data reveals distinct clustering and separation of samples from different locations and of fungal and bacteria communities (Figures 2A,B). PERMANOVA analysis (*p* ≤ 0.05) indicates a significant impact of location on both fungal and bacteria communities (*p* = 0.001), with a higher *R*^2^ for bacteria communities (Figure 2A). Analysis of group dispersion uniformity shows significant differences in the dispersion of bacteria communities between soil and rhizome stems (FG = 3.50, FT = 4.85, *p* = 0.001, after 999 permutations), as well as significant differences in the dispersion of fungal communities between soil and rhizome stems (FG = 3.17, FT = 3.21, *p* = 0.001, after 999 permutations). The dispersion differences between bacteria communities among locations are also significant (Table 3). PERMANOVA analysis suggests significant differences in both fungal and bacteria communities between PCH and soil samples, regardless of the origin (*p* = 0.001) (Table 3). Furthermore, significant differences exist between locations GS and GG, TS and TG for rhizosphere bacterial communities, and fungal and bacteria communities are clustered separately from and separated from several other locations (Figures 2A,B).

**Table 3:** Pairwise PERMANOVA (*pairwise.adonis*) comparisons for all combinations of soil and PCH (*p*-value < 0.05 in bold).

Pairwise PERMANOVA for consistency				
	Pairs	*F*. model	*R* ^2^	*p*-value
Fungi	GS vs GG	3.172	0.04335	**0.001**
	TS vs TG	3.2077	0.04382	**0.001**
Prokaryotes	GS vs GG	3.4977	0.04759	**0.001**
	TS vs TG	4.8457	0.06474	**0.001**

Regarding microbial communities within PCH, significant differences in the structure of both fungal and bacteria communities were observed among PCH samples from different locations (PERMANOVA, *p* ≤ 0.05) (Figures 2A,B). It is noteworthy that Subclass values range from 31.28 to 31.66%, with significant differences between the two major groups in Sichuan and Guangxi, indicating that there may be differences between the two microbial communities.

### 5.1 Heatmap analysis

**Figure 3:**
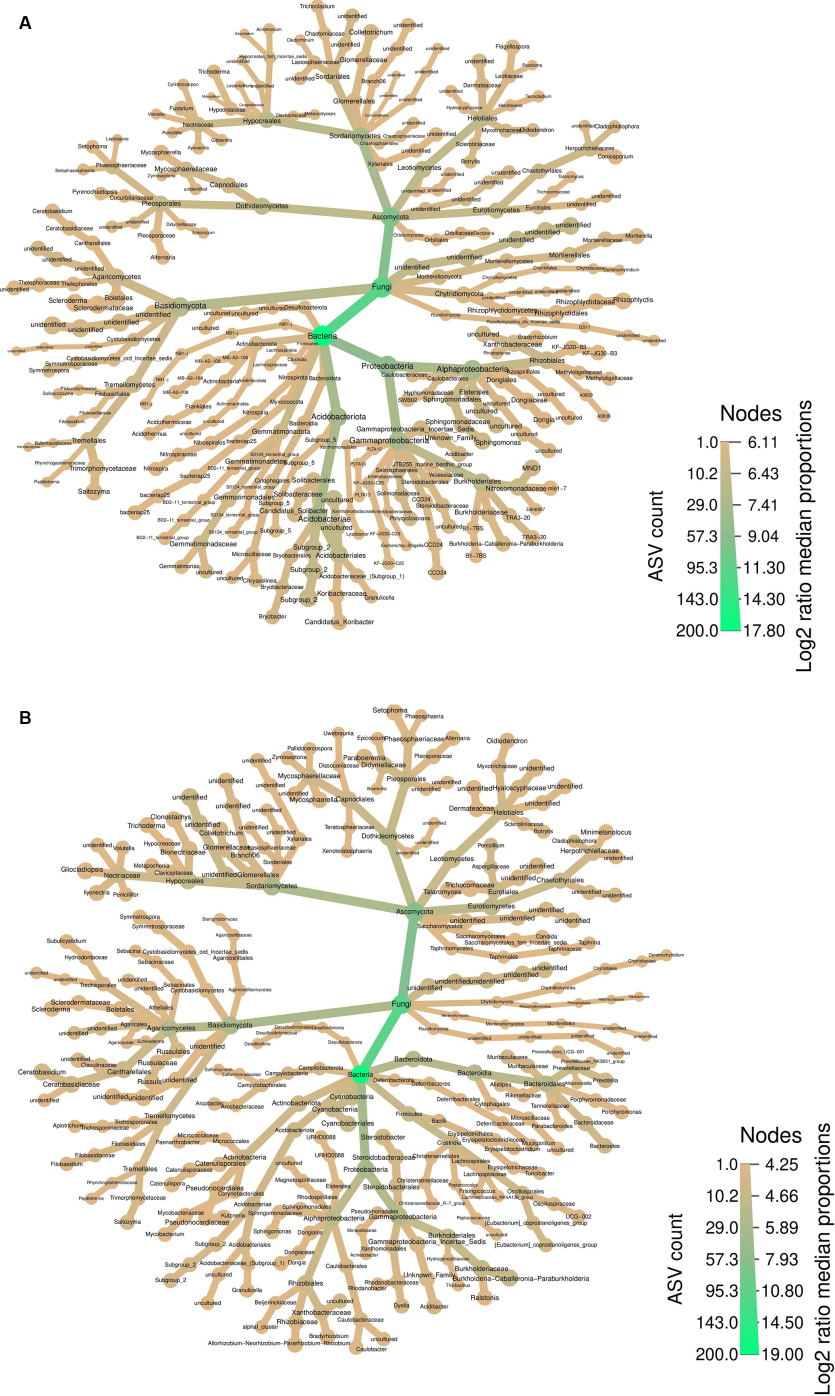
Differential Heatmaps, the differential heatmaps illustrate differences in species abundance at the family level between PCH rhizome **(A)** and the surrounding soil samples **(B)**. Colors represent log2 median ratios: green indicates significantly higher abundance of the taxa in PCH (after “FDR”correction, *p* ≤ 0.05), while light green indicates significantly higher abundance across all samples. Node size is proportional to the number of ASVs within each taxonomic group.

The heatmap provides a comprehensive perspective on the abundant taxa, summarizing the bacterial and fungal communities in both soil samples surrounding PCH rhizome (Figure 3A) and the rhizome themselves (Figure 3B). In both soil and rhizomes, *Ascomycota* and *Proteobacteria* are the most abundant fungal and bacterial phyla, respectively. The fungal community in the rhizome consists mainly of *Basidiomycota* and *Ascomycota*, with various orders and genera represented. Similarly, the bacteria community in the rhizome is dominated by *Proteobacteria*, *Bacteroidota*, *Firmicutes*, and *Actinobacteriota*, with diverse genera such as *Porphyromonas*, *Bacteroides*, *Burkholderia-Caballeronia-Paraburkholderia*, A*llorhizobium-Neorhizobium-Pararhizobium-Rhizobium*, and *Mycobacterium* (Figure 3A).

In the soil, the fungal community comprises *Basidiomycota* and *Ascomycota*, including *Cladophialophora*, *Flagellospora*, *Trichocladium*, *Acremonium*, *Fusarium*, *Mycosphaerella*, *Setophoma*, and *Saitozyma*, among others. The bacteria community in the soil is characterized by *Acidobacteriota*, *Proteobacteria*, and *Gammatimonadota*, with representatives like *Methyloigellaceae*, *Burkholderia-Caballeronia-Paraburkholderia*, *Candidatus-Koribacter*, *Bryobacter*, and *Gemmatimonas* (Figure 3B). In summary, the fungal community in PCH consists of the phyla *Basidiomycota* and *Ascomycota*, while the bacteria community consists of the phyla *Acidobacteriota*, *Proteobacteria*, *Bacteroidota*, *Firmicutes*, *Actinobacteriota*, and *Gammatimonadota*.

**Figure 4:**
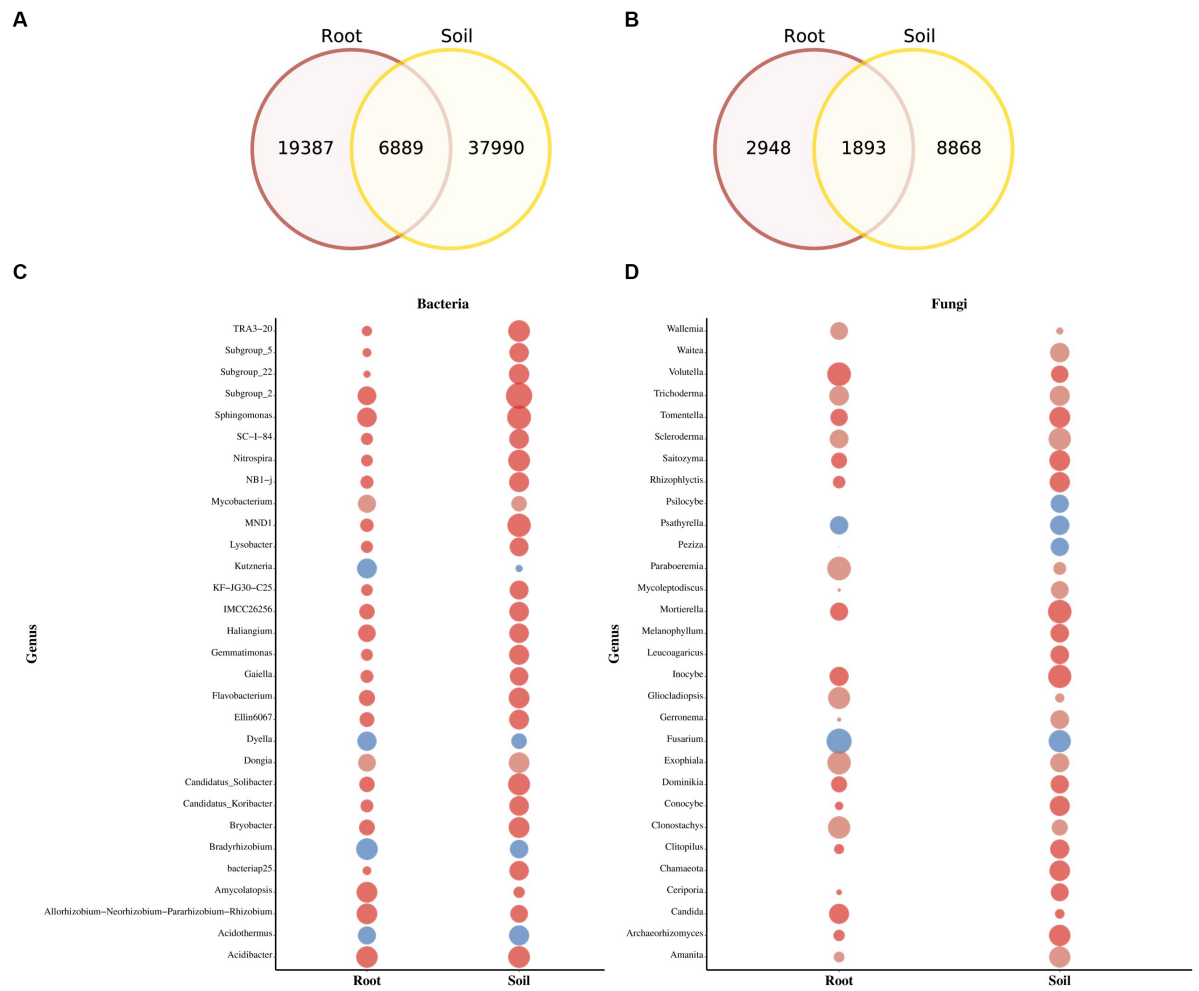
The figure depicts Venn diagrams showing the core and unique ASVs between soil and PCH in bacteria **(A)** and fungal **(B)** communities. The bubble plots display the relative abundance of bacteria **(C)** and fungal **(D)** genera in soil and PCH. The size of the bubble represents the percentage of relative abundance.

The Venn diagram highlights substantial differences between the bacterial and fungal ASVs in soil and rhizomes. Soil samples contain a large number of unique bacterial ASVs (8,868), while rhizome samples have 2,948 ASVs, with 1,893 shared between the two (Figures 4A,B). The relative abundance of fungal and bacteria taxa varies significantly between soil and PCH rhizome (Figures 4C,D). In soil samples, *Basidiomycota* (44.8%), *Ascomycota* (41.7%), *Agaricomycetes* (38.7%), *Sordariomycetes* (18.2%), and *Agaricales* (16.5%) dominate the fungal community. The most abundant bacterial phyla are *Proteobacteria* (42.0%), *Acidobacteriota* (23.3%), *Gammaproteobacteria* (22.6%), *Acidobacteria* (19.9%), *Alphaproteobacteria* (19.4%), *Burkholderiales* (13.2%), *Actinobacteriota* (9.2%), and *Bacteroidota* (7.3%). In PCH rhizome, *Ascomycota* (67.8%), *Basidiomycota* (26.1%), *Sordariomycetes* (18.2%), *Agaricomycetes* (30.1%), *Leotiomycetes* (21.8%), *Eurotiomycetes* (10.8%), *Dothideomycetes* (10.3%), *Hypocreales* (18.2%), *Helotiales* (13.0%), *Cantharellales* (11.5%), *Nectriaceae* (14.0%), *Ceratobasidiaceae* (11.2%), and *Hyaloscyphaceae* (9.1%) are the predominant fungal groups. The most abundant bacterial phyla include *Proteobacteria* (46.6%), *Actinobacteriota* (16.7%), *Cyanobacteria* (12.7%), *Bacteroidota* (9.8%), *Acidobacteriota* (4.7%), *Gammaproteobacteria* (26.4%), *Alphaproteobacteria* (21.5%), *Actinobacteria* (13.5%), Cyanobacteria (13.3%), and *Bacteroidia* (9.4%). Indicative species analysis indicates significant associations between bacterial ASVs from different locations and PCH rhizome and surrounding soil. Similar associations are observed for fungal ASVs (*p* < 0.05) (Table 2). Most taxonomic groups displayed in Figures 4C,D are matched with ASVs found in PCH rhizome, indicating corresponding fungal and bacterial communities in both soil and rhizome.

### 5.2 Relationship between morphological characteristics of PCH from different regions and microorganisms

**Figure 5:**
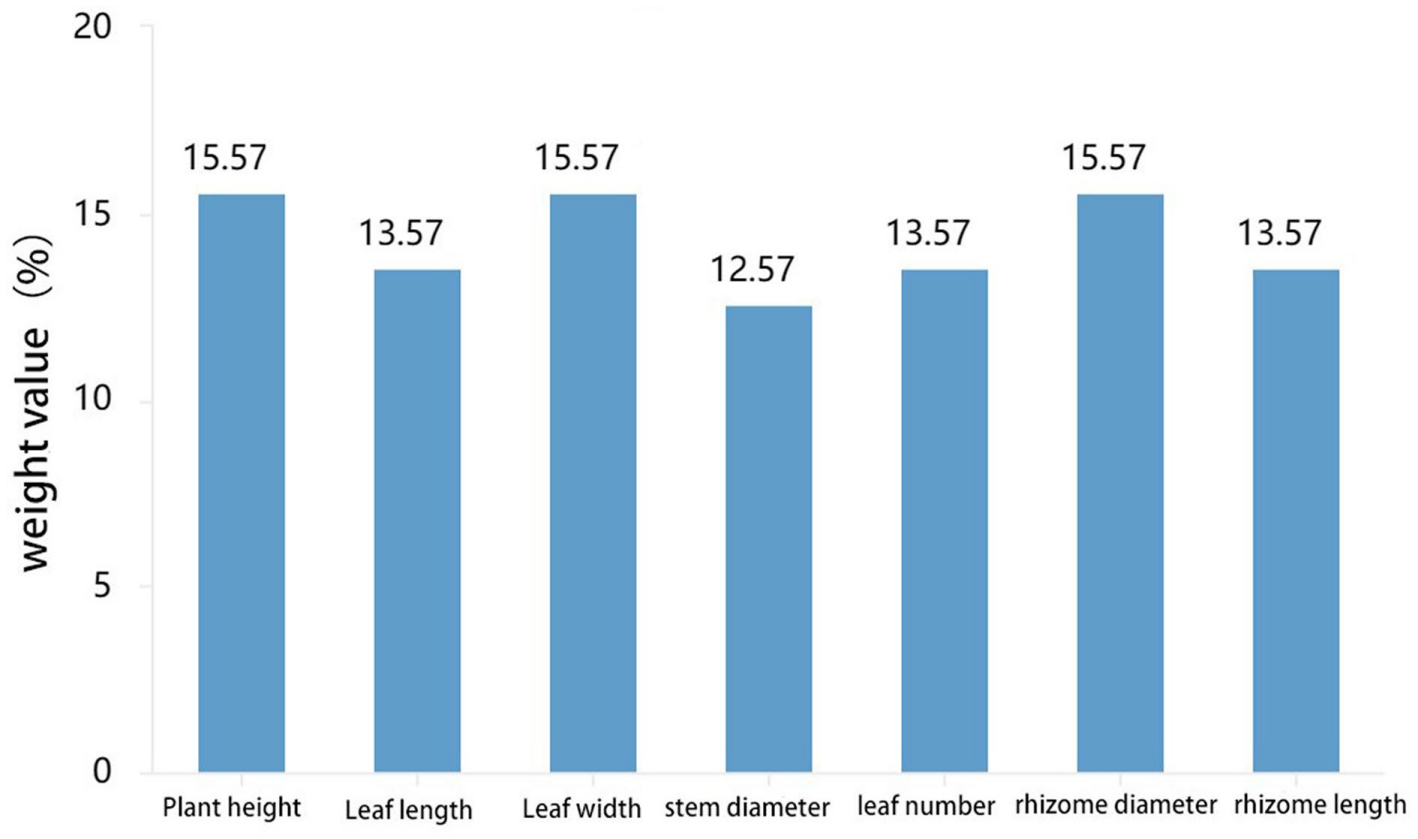
Analysis results of phenotypic weight of PCH.

Leaves are one of the most crucial organs during plant growth, while rhizomes are intuitive manifestations of the quality of certain medicinal plants. Phenotypic traits and the size of rhizomes reflect their adaptability to the ecological environment, serving as essential reference standards (Cheng et al., 2023). According to the SPSS weight analysis, plant height, leaf width, and rhizome diameter have high weight proportions in the morphological characteristics of PCH (as shown in Figure 5). Rhizomes promote growth through microbial interactions mediated by soil microbiota, and the core microbial community associated with rhizomes can influence their growth (Beals et al., 2022). Bacteria and fungi were subjected to correlation analysis with phenotypic indicators at the genus level using GraphPad Prism software, preliminarily screening out potentially related microorganisms (Chen et al., 2019). At the bacterial genus level, *Acidothermus* has consistently low abundance in all six locations in Sichuan but is present in all six locations in Guangxi, indicating a potential correlation with the origin of the sample (Xiong et al., 2022). Overall, *Sphingomonas* and *Candidatus-Solibcter* show an inverse relationship with plant height, while *Mortierella*, *Ceratobasidium*, and *Fusarium* show a positive correlation with rhizome diameter. *Sphingomonas* and *Candidatus-Solibcter* may act as height-suppressing bacteria, while *Nitrospira* and *Acidibacter* may be dominant bacterial groups associated with plant height (Quattrone et al., 2024).

**Figure 6:**
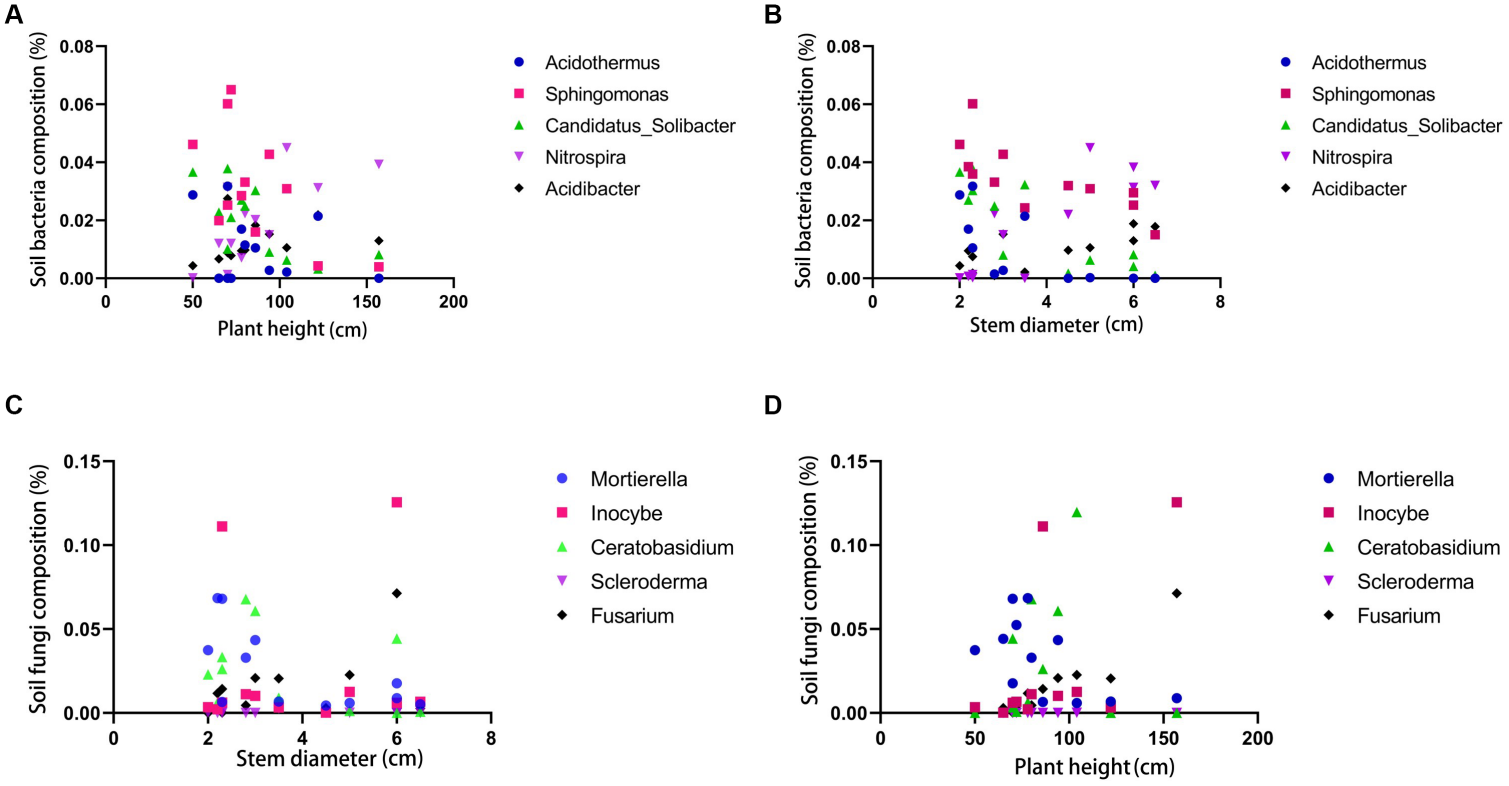
Correlation analysis between soil bacteria and plant height **(A)** and stem diameter **(B)**; correlation analysis between soil fungi and plant height **(C)** and stem diameter **(D)**.

The correlation analysis between rhizome diameter and bacteria reveals that *Acidothermus* has a high proportion in rhizome diameter, while *Sphingomonas* and *Candidatus-Solibcter* show an inverse correlation with rhizome diameter. *Nitrospira* and *Acidibacter* overall exhibit a positive correlation with rhizome diameter. *Acidothermus*, *Sphingomonas*, and *Candidatus-Solibcter* may act as rhizome-suppressing bacteria, while *Nitrospira* and *Acidibacter* may represent dominant bacterial groups associated with rhizome diameter. (Results analysis is shown in Figure 6).

### 5.3 Differential analysis of microbial communities in PCH from two regions

**Figure 7:**
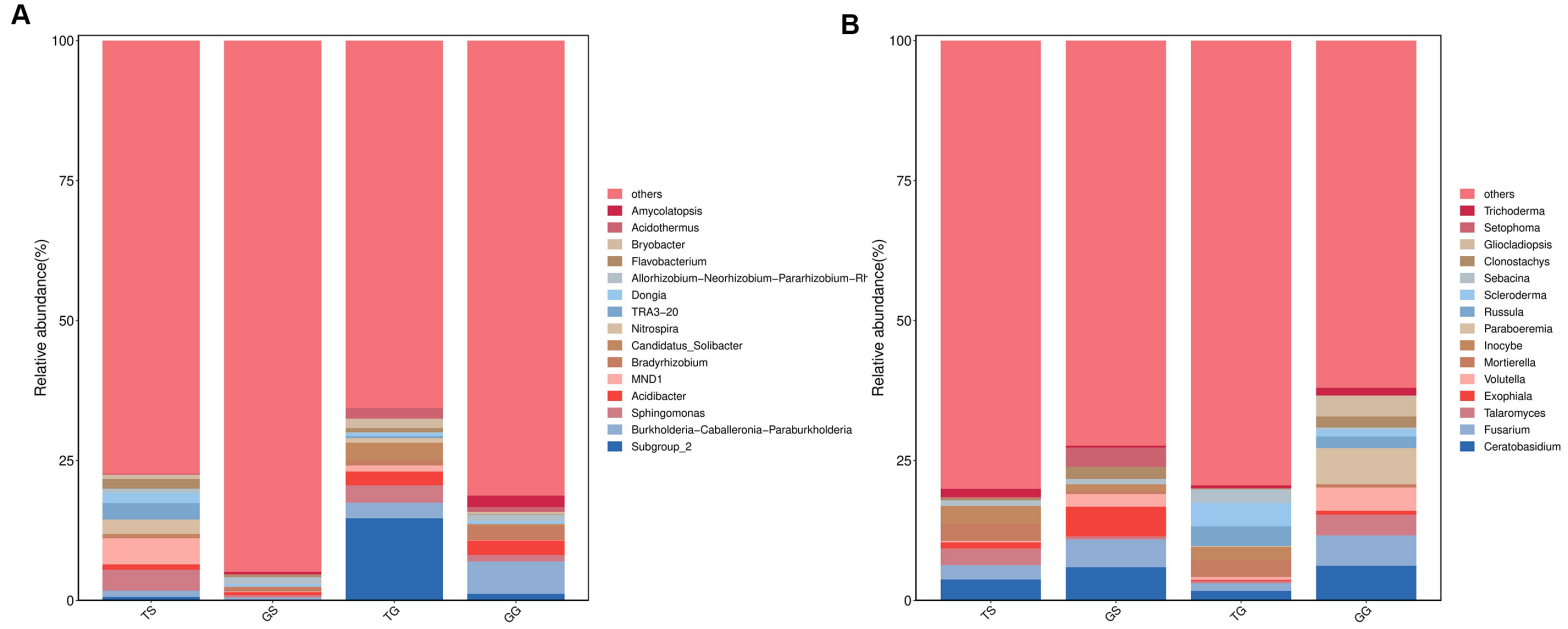
Comparative analysis of microbial communities (at the genus level) between Sichuan and Guangxi. Comparison of bacteria microorganisms in soil and rhizomes **(A)**, and fungal microorganisms in soil and rhizomes **(B)**.

Molecular variance analysis (pairwise. Adonis, PERMANOVA) yielded a *p*-value of 0.001 (*p* < 0.05), indicating a significant difference in microbial community composition between Sichuan and Guangxi province (Table 2). The bar graph (Figure 7) illustrates the genera of differentially abundant ASVs in the two regions. At the genus level of soil prokaryotes, compared to Sichuan, the genera *Subgruop-2*, *Burkholderia-Caballeronia-Paraburkholderia*, *Candidatus-Solibacter*, *Acidibacter*, *Bryobacter*, *Acidothermus*, and *Gemmatimonas* were significantly upregulated in Guangxi soil, while *Sphingomonas*, *MND1*, *Nitrospira*, *Flavobacterium*, *Dongia*, and *NB1-j* were downregulated. In rhizome prokaryotes, compared to Sichuan, the genera *Burkholderia-Caballeronia-Paraburkholderia*, *Bradyrhizobium*, *Acidibacter*, *Amycolatopsis*, *Sphingomonas*, *Steroidobacter*, *Dyella*, *Subgruop-2*, and *Mesorhizobium* were significantly upregulated, while *Allorhizobium-Neorhizobium-Pararhizobium-Rhizobium*, *Kutzneria*, *Prevotella*, *Lechevalieria*, and *Bacteroides* were downregulated. Shared bacteriagenera in both rhizomes and soil included *Subgruop-2*, *Burkholderia-Caballeronia-Paraburkholderia*, *Acidibacter*, and *Sphingomonas*.

At the genus level of soil fungal communities, compared to Sichuan, Guangxi soil had specific genera *Scleroderma*, *Russula*, *Archaeorhizomyces*, *Saitozyma*, and *Colletotrichum*, with high abundances of *Amanita* and *Tomentella*. Conversely, compared to Guangxi, Sichuan had specific genera *Rhizophlyctis* and *Chamaeota*, with higher abundances of *Ceratobasidium* and *Fusarium*. In rhizome fungal communities, compared to Sichuan, Guangxi rhizomes had specific genera *Paraboeremia*, *Gliocladiopsis*, *Russula*, and *Trichoderma*, with higher abundances of *Volutella* and *Talaromyces*. Conversely, compared to Guangxi, Sichuan rhizomes had specific genera *Setophoma*, *Candida*, and *Llyonecria*, with higher abundances of *Exophiala*, *Clonostachys*, *Mycosphaerella*, and *Tetracladium*. Shared fungal genera in both rhizomes and soil included *Ceratobasidium*, *Fusarium*, *Talaromyces*, and *Russula*.

### 5.4 Metabolic characteristics analysis of bacteria microbial communities

Tax4Fun was employed to predict the metabolic functions of bacterial communities in PCH. Tax4Fun utilized SILVA database results for classification, established an association matrix through BLASTN analysis, and predicted microbial community functions by linearly correlating SILVA classification results with KEGG database bacteria classification. A total of six primary metabolic pathways were annotated, encompassing metabolism, genetic information processing, environmental information processing, cellular processes, human diseases, and organismal systems. At the third level of KEGG metabolic pathways, Kruskal–Wallis tests were performed on each type of metabolic pathway, calculating the relative abundance among all samples from different regions. The results, based on the relative abundance percentages, were presented for rhizomes in Supplementary Figure S4. From the figure, it can be observed that the microbial samples are annotated to metabolic pathways (ko3088), biosynthesis of secondary metabolites (ko1990), microbial metabolism in diverse environments (ko1992), biosynthesis of amino acids (ko6147), carbon metabolism (ko0059), and ABC transporters (ko2004) with relatively low average proportions, all of which have a significant impact (*p* ≤ 0.05). Additionally, the metabolic pathways in the environmental information processing category, especially the metabolic pathways (ko3088), are the most abundant in the annotated microbial samples, (ABC transporters, ko2004) have the lowest proportion (See Table 3).

**Figure 8:**
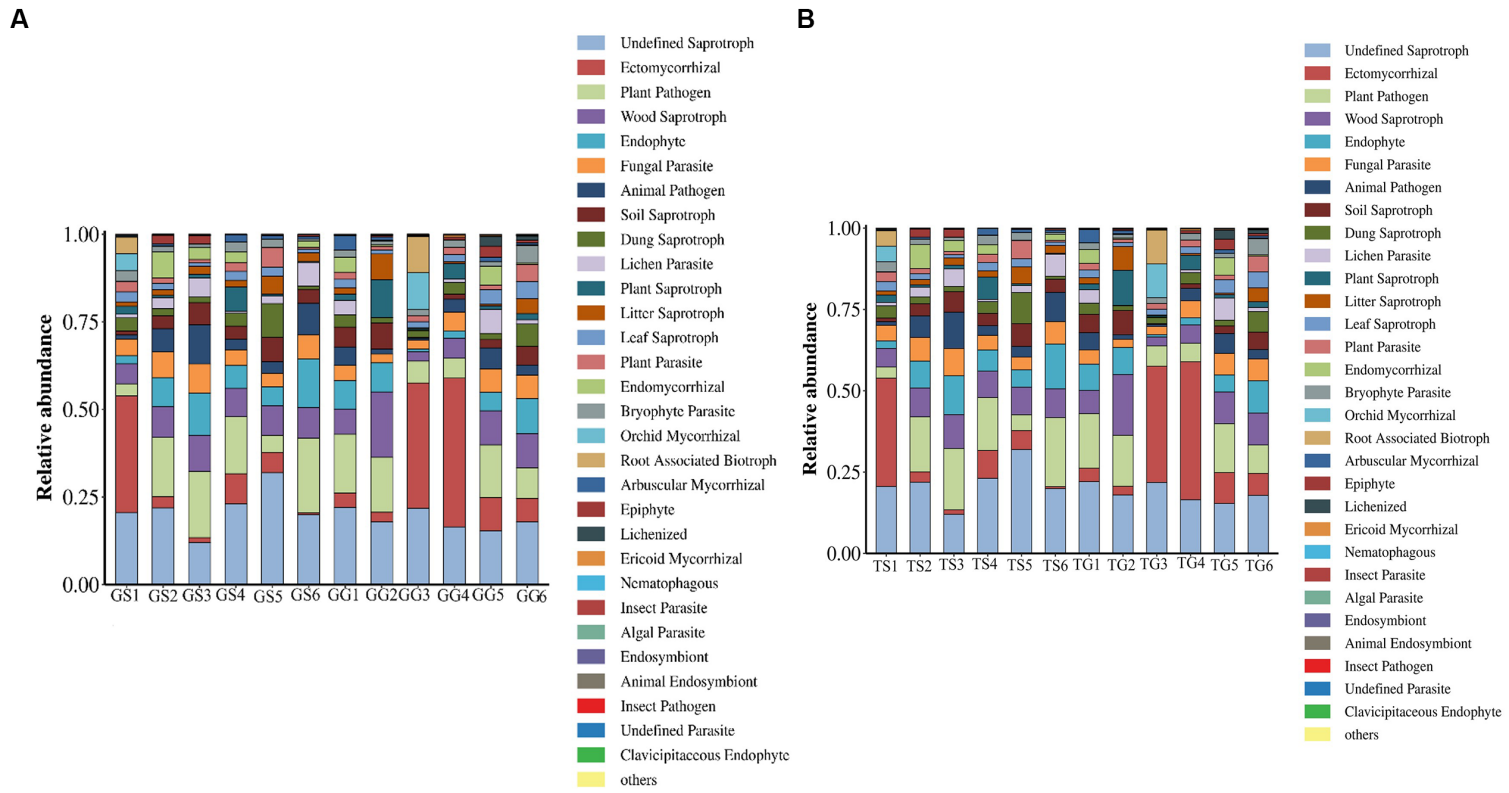
Fungal microbial communities in soil and rhizomes were grouped into Level-3 functional categories using Fungild, and a KEGG diagram of normalized average proportions was plotted.

Fungild was employed to predict the metabolic functions of fungal communities in PCH. Fungild first utilized the SILVA database for classification, established an association matrix through BLASTN analysis, and predicted microbial community functions by linearly correlating SILVA classification results with KEGG database fungal classification. A total of 30 KEGG metabolic pathways were annotated, including pathways related to ectomycorrhizal, plant pathogen, wood saprotroph, endophyte, fungal parasite, and plant saprotroph. Kruskal–Wallis tests were conducted for each type of metabolic pathway, calculating the relative abundance among all samples from different regions. Results, based on the relative abundance percentages, were presented for rhizomes in Figure 8. The graph illustrates those ectomycorrhizal fungi in the soil (TS1, TG3, TG4) and rhizomes (GS5) have the highest percentage, while plant pathogenic fungi in the soil (TS5, TS1) and rhizomes (GG3) have lower percentages. Wood saprotroph fungi in the soil (TG3) have a lower percentage.

### 5.5 The correlated analysis between total polysaccharides, saponins, and microbiota

The linear equations for the absorbance versus concentration of glucose and diosgenin are, respectively, expressed as *y* = 0.4820*x* − 0.1806 (*r* = 0.9994, linear range: 0.02000–0.4086 mg/mL) and *y* = 1.5043*x* + 0.0668 (*r* = 0.9999, linear range: 0.1740–1.4192 mg/mL). The contents of polysaccharides and saponins from different sources are shown in Table 4.

**Table 4:** Results of PCH polysaccharide and saponin content determination.

Sample ID	Polysaccharides (mg/g)	Saponins (mg/g)
S1	16.5887	0.2718
S2	19.0062	0.8626
S3	20.0593	1.4126
S4	16.9446	1.9183
S5	19.9139	2.3368
S6	16.3154	3.1058
G1	25.1446	1.6507
G2	18.4154	0.2828
G3	18.1149	0.8680
G4	21.1152	1.4572
G5	21.2032	2.0192
G6	27.1124	2.3897

To evaluate the quality of PCH, total polysaccharides and saponins are selected as quality assessment indicators. Given their significance as the main active components of PCH, these two constituents are chosen for quality evaluation (Wang et al., 2021; Zhang et al., 2024). Correlation analysis between the microbiota at the genus level and the chemical content from 12 different origins of PCH was conducted using GsraphPad Prism. Among the genus-level microbiota, the top 6 genera were selected based on their high abundance for further analysis. These genera are likely to play significant roles in microbial community structure and function due to their higher abundance, potentially influencing metabolic pathways and community dynamics. This preliminary screening aimed to identify potential microorganisms that may correlate with the quality of PCH.

**Figure 9:**
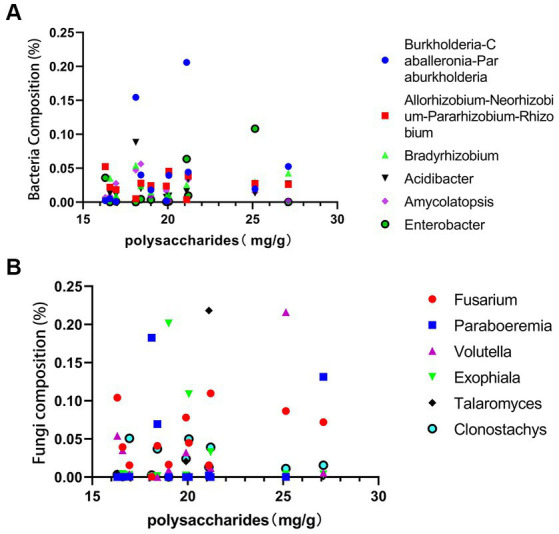
Correlation analysis between polysaccharides and bacterial abundance **(A)** and fungal abundance **(B)**.

As shown in the Figures 9A,B, total polysaccharide is highest in sample G6 from Guangxi and lowest in sample S6 from Sichuan. The fungi *Volutella* shows a direct proportionality to total polysaccharides, while *Exophiala* and *Clonostachys* exhibit an inverse proportionality to the polysaccharides content. *Fusarium* fungi demonstrate direct proportionality to both total polysaccharides and saponins content, whereas *Clonostachys* shows an inverse proportionality to saponins. Among bacteria, *Burkholderia-Caballeronia-Paraburkholderia*, *Acidibacter*, and *Amycolatopsis* show an inverse proportionality to PCH polysaccharides, while *Enterobacter* shows a direct proportionality. Similarly, *Burkholderia-Caballeronia-Paraburkholderia*, *Acidibacter*, *Amycolatopsis*, and *Enterobacter* show an inverse proportionality to PCH saponins.

## 6 Discussion

Previous studies have indicated that microorganisms play a metabolic role in species of the *Polygonatum* genus, encompassing functions such as energy metabolism, carbohydrate metabolism, and amino acid metabolism within the endophytic bacteria (Zhu et al., 2022; Xie et al., 2024). In this study, the microbial communities associated with PCH from 12 different locations in Sichuan and Guangxi provinces (including the rhizomes of PCH and the surrounding soil) were systematic identified and compared. The fungal and bacteria taxa in the rhizomes of PCH were analyzed and compared with those in the surrounding soil. Principal coordinate analysis (PCoA) and PERMANOVA analysis revealed distinct location-related effects on the classification structure of fungal and bacteria communities, with samples from Sichuan and Guangxi clustering in different directions according to location. Specifically, samples from Sichuan’s Li County formed a separate cluster, distinct from other locations. This finding suggests that specific soil characteristics and altitudinal factors from different origins may influence the microbial classification structure. However, it is noteworthy that the microbial communities associated with PCH converged across several microbial community classifications. Research on soil microbial communities supports the idea that different microbial communities can impact the growth and development of medicinal plants (Zhang et al., 2020b; Wang et al., 2023; Lü et al., 2023b). Nevertheless, the microbial communities associated with PCH focused on multiple microbial community classifications. Our data on PCH align with the findings of Liu and Wang (Bai et al., 2020), where the microbial communities of most PCH species were similar regardless of geographical origin or microbial diversity. Additionally, a more in-depth analysis of soil microbial differences was not conducted in this study. Soil samples were taken from a depth of approximately 5–10 cm around the rhizomes, and previous research has shown that comparing depths of 0 cm, 40 cm, 100 cm, and 200 cm revealed no detectable differences in α-diversity or β-diversity. This suggests that the impact of PCH on the soil microbial community is minimal, allowing us to conclude that depth and microbes are not strongly correlated in this experiment (Giorgio et al., 2023).

Many microorganisms in the soil and rhizomes exhibit statistical coexistence. Specifically, there are 1,893 shared fungi and 6,889 shared bacteria between the soil and rhizomes, indicating a higher abundance of bacterial communities in both the rhizomes and soil compared to fungal communities. Among the top 30 microbial groups selected in the bubble chart, certain fungal taxa such as *Waitea*, *Psilocybe*, *Peziza*, *Melanophyllum*, *Leucoagaricus*, and *Chamaeota* were not expressed in the rhizomes but were present in the soil. In contrast, some bacterial taxa, including *Gliocladiopsis*, *Candida*, *Amycolatopsis*, and *Kutzneria*, exhibited higher expression levels in the rhizomes compared to the soil. It is noteworthy that these microbial groups, particularly those with higher expression in the rhizomes, such as *Gliocladiopsis*, *Candida*, *Amycolatopsis*, and *Kutzneria*, have been reported in the literature as beneficial microorganisms with plant growth-promoting effects (Shi et al., 2023). Therefore, these microbes could be considered as beneficial organisms in the rhizomes, potentially promoting the growth and development of the rhizomes.

The interaction between plants and microorganisms promotes plant growth, playing a crucial role in the intricate and complex interactions occurring in the rhizosphere and surrounding areas. Microorganisms, including bacterial, fungal, and viral communities, are key contributors to enhancing plant health and overall growth. Soil microorganisms, such as plant growth-promoting bacteria and arbuscular mycorrhizal fungi, play a significant role in plant growth by synthesizing plant growth hormones such as auxins, cytokinins, and gibberellins, thereby facilitating rhizome development, shoot elongation, and overall growth (Aqeel et al., 2023). The *Acidobacteriota* and *Basidiomycota* have been identified as beneficial strains for the quality of PCH. Tests have been conducted on these strains, and disease-resistant agents and microbial fertilizers have been developed accordingly (Sliti et al., 2024). Based on the discussed microbial-plant trait associations, there may be a certain correlation between rhizome characteristics and microorganisms, potentially participating in the regulation of PCH plant traits. Bacteria such as *Sphingomonas* and *Candidatus-Solibacter* may exhibit a negative correlation with plant growth, warranting further exploration of the microbial regulation of PCH plant growth. The bacterial genus *Sphingomonas* is distributed in both Guangxi and Sichuan, present in both rhizomes and soil. *Sphingomonas* bacteria are known for their ability to utilize various organic compounds to synthesize indole-3-acetic acid (IAA), enhancing nutrient conditions and IAA production to promote plant growth (Khan et al., 2014). This indicates that PCH harbors numerous beneficial bacteria that can protect plants from environmental stress. Most core bacterial groups exhibit rich metabolic diversity, producing nutrients under nutrient-poor soil conditions, providing a material foundation for plant growth and development (Asaf et al., 2017).

Research has revealed the crucial role of rhizosphere microbial diversity in influencing biomass, with plant growth showing a positive correlation with certain rhizosphere bacteria (Xiao et al., 2017). The soil microbial community forms a fundamental ecosystem that hosts a multitude of biological hosts. Microorganisms play a vital role in the health and performance of plants, and the content of any soil microbial community directly determines plant productivity. In a sense, the richer the microbial community, the healthier the plants. Due to this reason, the interaction between plants and soil microbial communities has become a significant process, and multiple factors shape the function and composition of this microbial community (Gachara et al., 2023). However, in recent years, the stability of soil microbial communities has undergone significant changes, especially with the escalating challenges posed by climate change and global food shortages. The severe harm caused to these microbial communities has primarily catalyzed the emphasis and occurrence of anthropogenic stressors related to plants, thereby negatively impacting plant growth and performance. The introduction discusses the fundamental role of soil microbial communities in driving plant growth while outlining the adverse effects of drought, high temperatures, salt stress, and climate change on plant growth. As mentioned earlier, soil microbial communities colonize all accessible parts of plant tissues, endowing host plants with various advantages, including nutrient absorption, tolerance, growth promotion, enhanced performance, and resistance to pathogens. ABC transporters are widely involved in responding to abiotic stresses. Studies have shown that BC transporters are upregulated under salt and drought stress (Kou et al., 2024). However, ABC transporters have the lowest proportion in Tax4Fun, indicating that PCH is less affected by abiotic factors such as drought, increased salinity, and heavy metal pollution. The relative abundance of TS1 metabolism functions significantly decreases, with polysaccharides and saponins being low in content in S1 production areas, indicating an impact on the accumulation and synthesis of polysaccharides and saponins.

Xiao discovered a positive correlation between rhizobia (including *Flavisolibacter*, *Luteimonas*, and *Geodermatophilaceae*) and the growth of leguminous plants (Xiao et al., 2017). Their research suggested that plants, rhizospheric microbial communities, and soil are in constant communication through the exchange of nutrients and signals. Bubble and Venn diagrams displayed the richness of *Alphaproteobacteria* and *Proteobacteria* (belonging to rhizobia) among bacterial classifications, both in rhizomes and soil, where they played a dominant role in the bacterial community. As for fungal classifications, *Ascomycota* and *Basidiomycota* were the most abundant in rhizomes and soil, encompassing the majority of fungi in these two phyla, playing a crucial role in the biodiversity of the fungal kingdom (Schoch et al., 2009). GraphPad Prism analysis revealed a correlation between microbial communities and phenotypic traits. At the genus level, bacteria such as *Nitrospira* and *Acidibacter*, and fungi such as *Mortierella*, *Ceratobasidium*, and *Fusarium*, were found to potentially promote plant height and rhizome stem diameter. This finding supports existing literature reporting that rhizospheric microbial communities and soil are in constant communication through nutrient and signal exchange, with microbial communities positively correlated with plant growth (Jacoby et al., 2017). Furthermore, the dominant bacterial groups associated with advantageous phenotypic traits belonged to the *Proteobacteria* and *Acidobacteriota* phyla, encompassing the majority of bacterial communities in PCH. The dominant fungal groups associated with advantageous phenotypic traits belonged to the *Ascomycota*, *Basidiomycota*, and *Moritierellomycota* phyla, including the majority of fungal communities in PCH.

This study identified differential bacteria in PCH, and *Gemmatimonadaceae* was identified as a major differential bacterium in the rhizosphere microbial community of healthy *Polygonatum kingianum* Coll.et Hemsl (PKH). *Pseudoflavonifractor*, a member of *Gemmatimonadaceae*, showed higher abundance in the rhizosphere soil of PKH suffering from rhizome rot disease. However, when compared with microbial communities from 12 collection points, *Pseudoflavonifractor* associated with diseased rhizomes was not detected, indicating that samples from the 12 collection points were all healthy (Cao et al., 2023). Additionally, the microbial community of American ginseng with different growth ages had some impact but did not alter the core microbial community (Suárez et al., 2023). Future research should focus on further validating the selected dominant microbial communities associated with PCH. Investigating the relationships between these microorganisms and PCH growth, as well as determining whether specific microbial taxa are positively or negatively correlated with PCH yield, could serve as another variable for assessing the cultivation and management of PCH. To accomplish this, we plan to conduct isolation and inoculation experiments on the selected core microorganisms to validate their effects.

According to the heatmap, *Proteobacteria*, *Acidobacteria*, and *Firmicutes* are abundant bacterial phyla in the soil and rhizomes of PCH. Detection of these phyla suggests their involvement in phosphorus cycling, nitrogen cycling, plant growth promotion, or soil remediation. Moreover, their abundance is positively correlated with the total nitrogen content in the soil (Hegyi et al., 2021), indicating their promotion effect during the growth of PCH rhizomes. Additionally, it is noteworthy that *Firmicutes* and *Bacteroidota* have low abundance in the soil but show a significant increase in the rhizomes. *Firmicutes* and *Bacteroidota* are known for their ability to suppress pathogenic microbes and induce systemic resistance, playing a crucial role in soil organic matter decomposition (Huang et al., 2023; Velmurugan et al., 2023). This observation suggests that these bacterial phyla in PCH may possess disease resistance and soil organic matter decomposition capabilities.

In both the soil and rhizomes, fungi are composed of the *Ascomycota* and *Basidiomycota*. *Ascomycota* includes the classes *Eurotiomycetes*, *Leotiomycetes*, *Sordariomycetes*, and *Dothideomycetes*, while *Basidiomycota* comprises the class *Agaricomycetes*. These are the most abundant fungal taxa shared between the rhizomes and soil. Interestingly, although the presence of *Ascomycota* and *Basidiomycota* in PCH soil and rhizomes has been previously reported, the presence of *Eurotiomycetes*, *Leotiomycetes*, and *Agaricomycetes* classes in PCH soil has not been documented before. Research indicates that *Ascomycota* and *Basidiomycota*, belonging to the phylum *Dikarya*, are widely distributed fungal groups in many soil environments and represent dominant fungal phyla in soils (Zhang et al., 2020a). *Sordariomycetes* and *Dothideomycetes* classes have been identified as dominant fungal groups in PCH (Tian et al., 2023), suggesting their essential role in the development and growth of PCH.

As is widely recognized, the interactions between fungi and bacteria play a crucial role in the functioning of many ecological systems. They fundamentally regulate the behavior of each other and play important roles in ecosystems. Interactions between fungi and bacteria can be highly specific, where the same ectomycorrhizal fungus can stimulate bacterial growth under specific conditions (Hannula et al., 2019; Berrios et al., 2023). In the soil beneath PCH rhizomes, the structure of the bacterial community displays a rich diversity, with bacterial genera being more abundant than fungal genera. It is hypothesized that the colonization of microbial taxa on PCH begins in the soil, potentially serving as their natural source. These microorganisms then migrate inward into the rhizomes of PCH, undergoing selection and colonization before PCH growth. In this context, exogenous microorganisms may find more favorable growth conditions (Lü et al., 2023a; Lavallee et al., 2024).

PCH polysaccharides and saponins are primary active components and crucial indicators for evaluating the quality of PCH. *Exophiala* and *Clonostachys* contribute to the decrease in PCH polysaccharides content, while *Enterobacter* may participate in the synthesis of PCH polysaccharides, thereby increasing their content. Previous studies have shown that *Fusarium* significantly increases the content of polysaccharides and other metabolites (Li et al., 2023). *Acidibacter* can promote the synthesis and accumulation of terpenoid compounds and also has a certain impact on polysaccharide compounds (Ren et al., 2023). This study further confirms the close relationship between the content of PCH polysaccharides and saponins and these bacteria and fungi. Microorganisms play an essential role in regulating metabolic pathways and the synthesis and accumulation of metabolites, potentially providing new directions and possibilities for the synthesis of useful substances within organisms.

## 7 Conclusion

This study discovered diverse fungal and bacterial communities in the rhizomes of PCH and the surrounding soil, with higher bacterial diversity. Four core bacteriamicrobial genera were found in both soil and rhizome in Sichuan and Guangxi, namely *Subgroup-2*, *Burkholderia-Caballeronia-Paraburkholderia*, *Acidibacter*, and *Sphingomonas*. Guangxi province had three unique fungal genera, namely *Scleroderma*, *Russula*, and *Gliocladiopsis*, while Sichuan province had only one unique fungal genus, *Chamaeota*. This difference may be a significant environmental factor contributing to the quality variation of PCH between Guangxi and Sichuan. Specific microbial taxonomic groups play a crucial role in the geographical authenticity of medicinal herbs, closely associated with the quality of PCH, highlighting their importance in medicinal plant cultivation management. Further research will focus on the intervention of unique microorganisms in PCH cultivation, exploring the correlation between specific microbial communities and quality, providing scientific data for the selection of suitable habitats, optimization of cultivation, and improvement of product quality for PCH cultivation locations.

## Data availability statement

The datasets presented in this study can be found in online repositories. The names of the repository/repositories and accession number(s) can be found below: https://www.ncbi.nlm.nih.gov/, PRJNA1102658 and PRJNA1102536.

## Author contributions

LY: Writing – original draft, Writing – review & editing. QY: Data curation, Resources, Writing – original draft. JW: Investigation, Methodology, Writing – review & editing. YW: Formal analysis, Investigation, Writing – original draft. WJ: Methodology, Resources, Software, Writing – review & editing. ZY: Investigation, Resources, Writing – original draft. ZZ: Formal analysis, Methodology, Project administration, Supervision, Writing – original draft, Writing – review & editing.
