# Supplementary material for: Quality analysis and function prediction of soil microbial communities of Polygonatum cyrtonema in two indigenous-origins
Source: Front Microbiol. 2024 May 31;15:1410501. doi: 10.3389/fmicb.2024.1410501 (PMC11176499; doi:10.3389/fmicb.2024.1410501)
Supplement: Supplementary file 1 [file Image_1.pdf]

Figure S1

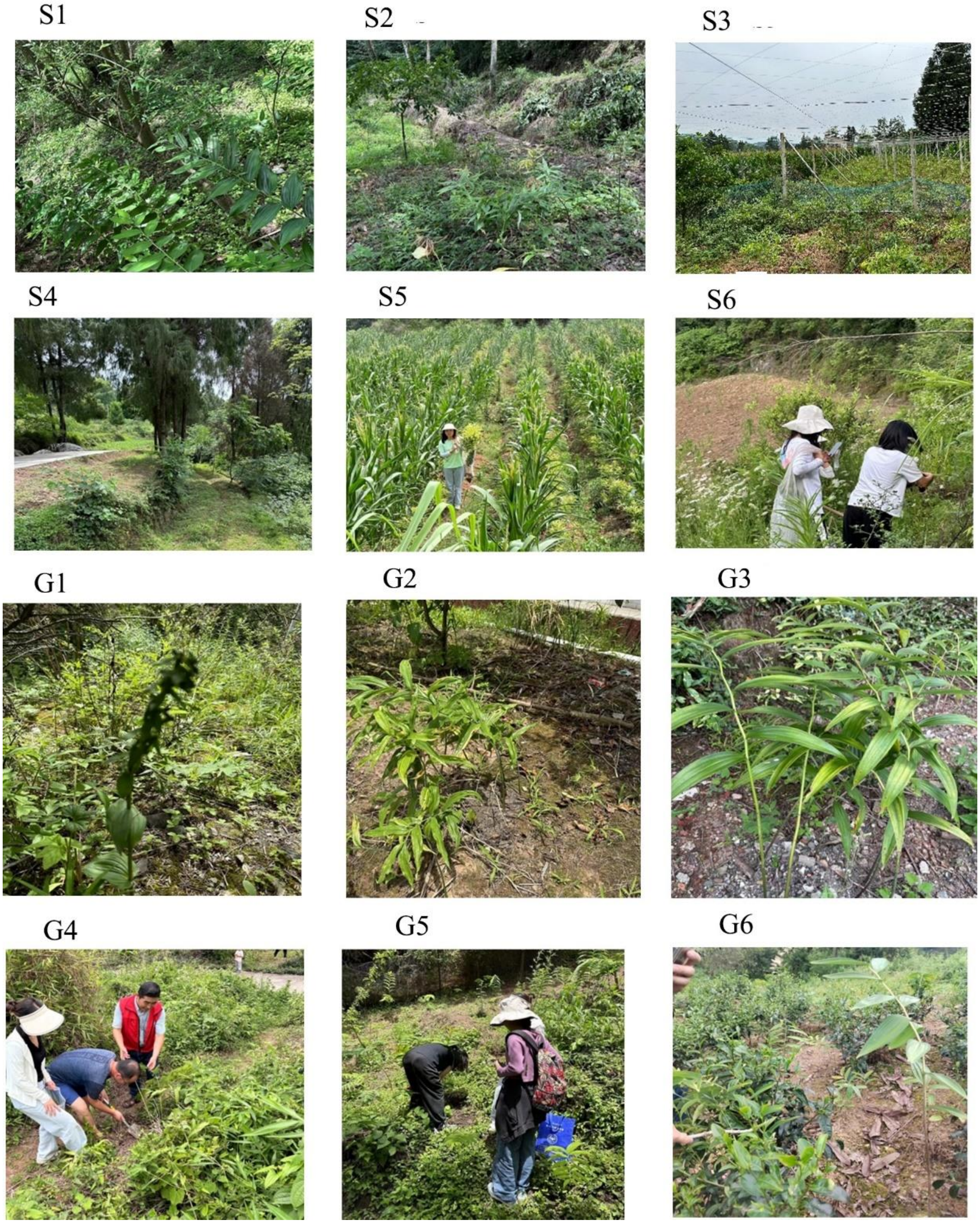

Figure S1 Cultured *Polygonatum cyrtonema* Hua (PCH) in different habitats in Sichuan and Guangxi province
