# Supplementary material for: Quality analysis and function prediction of soil microbial communities of Polygonatum cyrtonema in two indigenous-origins
Source: Front Microbiol. 2024 May 31;15:1410501. doi: 10.3389/fmicb.2024.1410501 (PMC11176499; doi:10.3389/fmicb.2024.1410501)
Supplement: Supplementary file 2 [file Image_2.pdf]

Figure S2

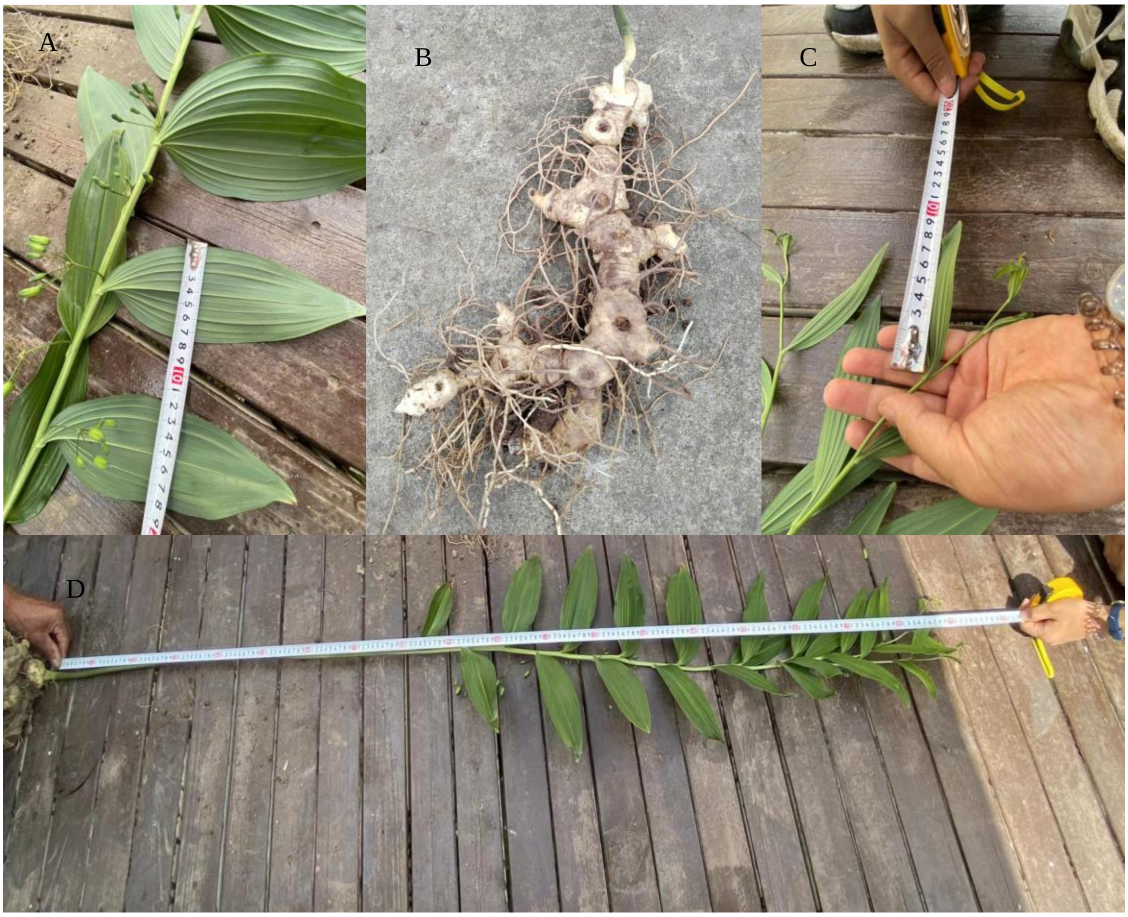

Figure S2 Measurement of phenotypic traits in PCH (A. Leaf width, B. Rhizome C. Leaf length, D. Plant height)
