# Supplementary figures and images for: Quality analysis and function prediction of soil microbial communities of Polygonatum cyrtonema in two indigenous-origins
Source: Front Microbiol. 2024 May 31;15:1410501. doi: 10.3389/fmicb.2024.1410501 (PMC11176499; doi:10.3389/fmicb.2024.1410501)

Figure S3

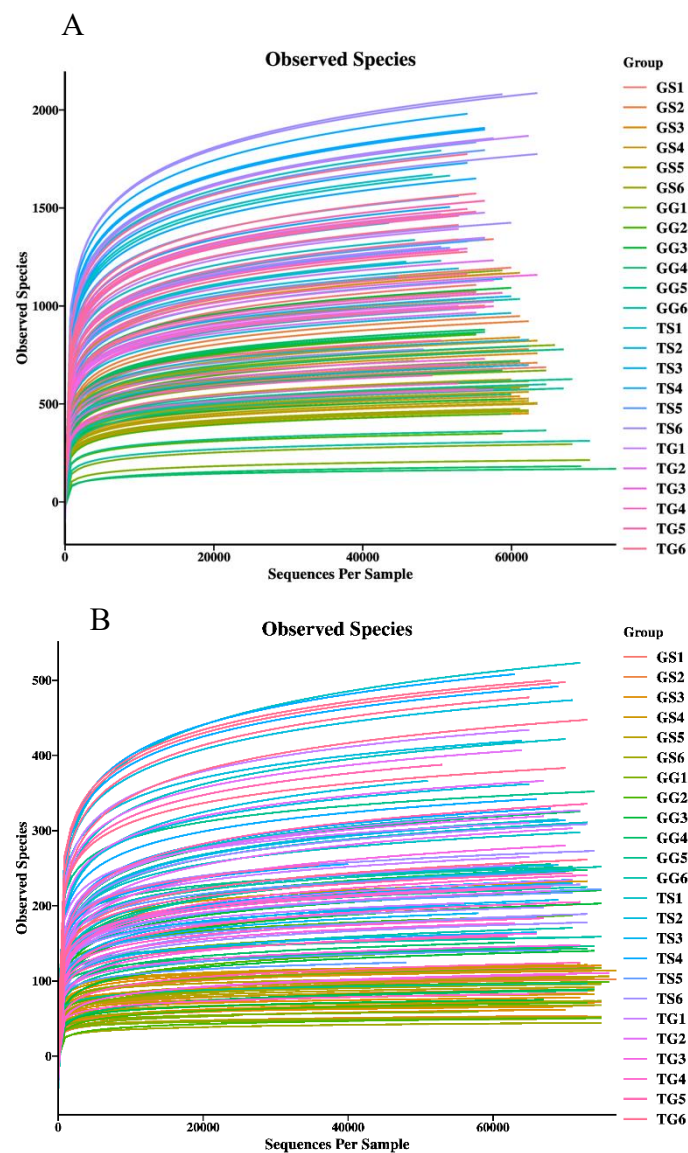

Figure S3 Rarefaction curve of bacteria(A) and fungi(B) in rhizome and soil of PCH

Supplement: Supplementary file 3 [file Image_3.pdf]
