# Supplementary material for: Quality analysis and function prediction of soil microbial communities of Polygonatum cyrtonema in two indigenous-origins
Source: Front Microbiol. 2024 May 31;15:1410501. doi: 10.3389/fmicb.2024.1410501 (PMC11176499; doi:10.3389/fmicb.2024.1410501)
Supplement: Supplementary file 4 [file Image_4.pdf]

Figure S4

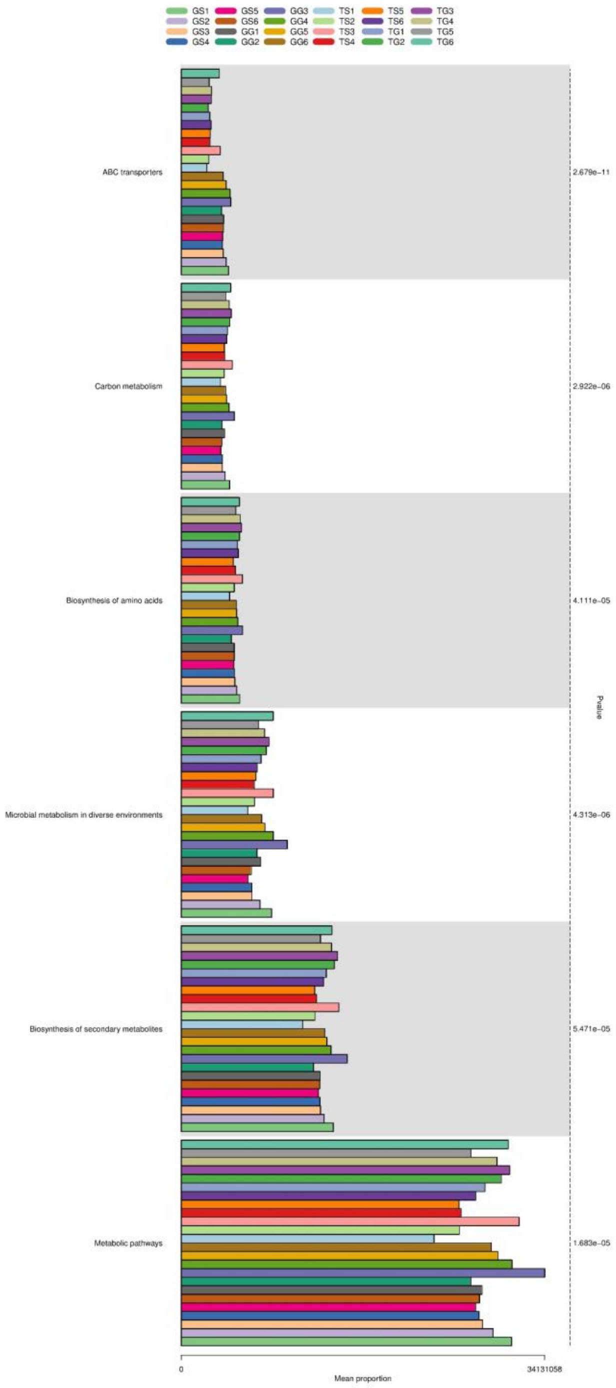

Figure S4 Bacterial microbial communities in soil and rhizomes were grouped into Level-3 functional categories using Tax4Fun, and a KEGG diagram of normalized B average proportions was plotted
